# A review of COVID-19 vaccines in development: 6 months into the pandemic

**DOI:** 10.11604/pamj.2020.37.124.24973

**Published:** 2020-10-05

**Authors:** Merlin Sanicas, Melvin Sanicas, Doudou Diop, Emanuele Montomoli

**Affiliations:** 1 Centre de Recherche en Cancérologie de Marseille, Université Aix-Marseille, Marseille, France,; 2 Clinical Development, Takeda Pharmaceuticals International AG, Zurich, Switzerland,; 3 PATH, Dakar, Sénégal,; 4 Department of Molecular and Developmental Medicine, University of Siena, Siena, Italy,; 5 VisMederi Life Science Research, Siena, Italy

**Keywords:** Coronavirus, COVID-19, pandemic, vaccine development

## Abstract

The advent of the COVID-19 pandemic and the dynamics of its spread is unprecedented. Therefore, the need for a vaccine against the virus is huge. Researchers worldwide are working around the clock to find a vaccine. Experts estimate that a fast-tracked vaccine development process could speed a successful candidate to market in approximately 12-18 months. The objective of this review was to describe the coronavirus vaccines candidates in development and the important considerations. The review was conducted through a thematic analysis of the literature on COVID-19 vaccines in development. It only included data until the end of June 2020, 6 months after the emergence of the COVID-19. Different approaches are currently used to develop COVID-19 vaccines from traditional live-attenuated, inactivated, subunit vaccines, to more novel technologies such as DNA or mRNA vaccines. The race is on to find both medicines and vaccines for the COVID-19 pandemic. As with drugs, vaccine candidates go through pre-clinical testing first before they go through the three phases of clinical trials in humans. Of the over 130 vaccine candidates, 17 are in clinical trials while others are expected to move to clinical testing after the animal studies.

## Introduction

Coronavirus is spreading around the world. As of July 12^th^, 2020, more than 12,552,736 cases of COVID-19 have been reported in over 216 countries and territories, resulting in 561,617 deaths [1, 2]. The virus spreads easily and most of the world's population is still vulnerable to it. It is therefore, of paramount importance to get a vaccine that can stop the spread of the virus. Researchers are working hard to control the pandemic. We are only over 6 months after the current outbreak was first reported from Wuhan, China yet the virus SARS-CoV-2 has been identified, sequenced, and shared to the whole world. This is unprecedented for a new disease. Rapidly advancing potential vaccines is critical to stemming the virus´s devastating impact on human health and the global economy. A vaccine would provide some protection by training people's immune systems to fight the virus so they should not become sick. Besides the anticipated health benefits from a coronavirus vaccine, there are several impacts on economic and social aspects. This would allow lockdowns to be lifted more safely, and social distancing to be relaxed. The objective of this review was to describe the coronavirus vaccines candidates in development and the important considerations.

## Methods

This review was conducted through a thematic analysis of the literature on COVID-19 vaccines in development. The review is conceptual and focuses on the WHO COVID-19 vaccines landscape, clinicaltrials.gov, media reports, and the respective websites of companies reported to be working on a COVID-19 vaccine. The review only included data until the end of June 2020, 6 months after the emergence of the novel coronavirus, SARS-CoV-2.

## Current status of knowledge

**Figure 1:**
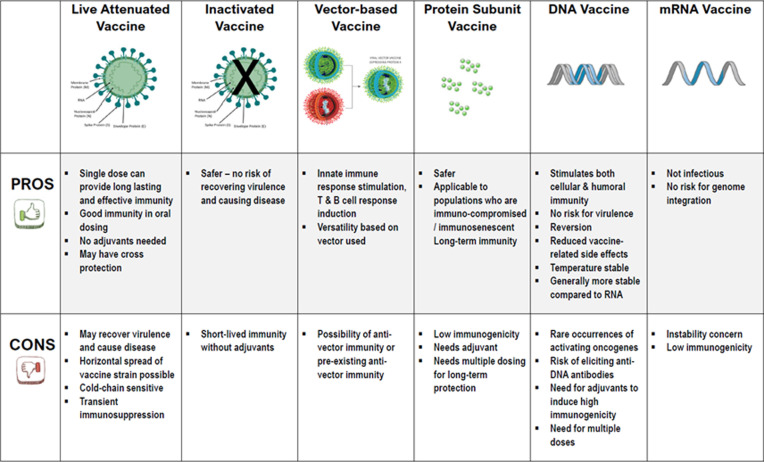
different vaccine approaches-their advantages and disadvantages

There are different types of vaccines in development for COVID-19. The pipeline includes over 130 candidates in development and as of end June 2020, 17 are already in various phases of clinical development, while the others are in preclinical development. Each of the different vaccine platforms available, traditional, or novel, is currently being explored. The World Health Organization (WHO) landscape of COVID-19 vaccine candidates (19 June 2020) lists 136 vaccine candidates [3]. Researchers striving to develop a coronavirus vaccine are working with different approaches, all with their respective advantages and disadvantages (Figure 1). Live attenuated vaccine [4, 5]; inactivated vaccine [5]; vector-based vaccine [6]; protein subunit vaccine [7]; DNA vaccine [6-11] and mRNA vaccines [6]. The sections below will discuss these different vaccine approaches. A summary of all the vaccine candidates currently in clinical trials is provided in Table 1.

**Table 1:** all COVID-19 vaccine candidates in clinical trials (as of 19 June 2020)

Phase	Name	Type	N	Age (years)	Randomized	Design	Location	Start date	End date	Study Number	Status
I	Cansino Ad5-nCoV	Non-replicating viral vector	108	18-60	No	Open-label, dose-finding	China	16/03/2020	30/12/2020	ChiCTR2000030906/ NCT04313127	Active, not recruiting
I	Moderna mRNA-1273	RNA	155	18-55	No	Open-label, dose-finding	USA	16/03/2020	22/11/2021	NCT04283461	Recruiting
I	Inovio INO-4800	DNA	120	≥18	No	Open-label, dose-finding	USA	03/04/2020	31/07/2021	NCT04336410	Recruiting
I/II	WIBP vaccine	Inactivated	1264	≥6	Yes	Double-blind, dose-finding	China	11/04/2020	10/11/2021	ChiCTR2000031809	Not yet recruiting
II	Cansino Ad5-nCoV	Non-replicating viral vector	508	18-60	Yes	Double-blind	China	12/04/2020	31/01/2021	NCT04341389	Active, not recruiting
I/II	Sinovac vaccine	Inactivated	744	18-59	Yes	Double-blind, dose-finding	China	16/04/2020	13/08/2020	NCT04352608	Recruiting
I/II	BioNTech BNT162	RNA	200	18-55	No	Open-label, dose-finding	Germany	23/04/2020	31/08/2020	NCT04380701	Recruiting
I/II	Oxford ChAdOx1	Non-replicating viral vector	1090	18-55	Yes	Single-blind	UK	23/04/2020	31/05/2021	NCT04324606	Active, not recruiting
I/II	BioNTech BNT162	RNA	7600	18-55	Yes	Observer-blind, dose-finding	USA	29/04/2020	28/06/2021	NCT04368728	Recruiting
I	Symvivo bacTRL-Spike	Other	84	19-55	Yes	Observer-blind, dose-finding	Canada	30/04/2020	31/08/2021	NCT04334980	Not yet recruiting
I/II	Cansino Ad5-nCoV	Non-replicating viral vector	696	18-84	Yes	Double-blind, dose-finding	Canada	01/05/2020	31/03/2021	NCT04398147	Not yet recruiting
II/III	Oxford ChAdOx1	Non-replicating viral vector	10260	≥5	Yes	Single-blind	UK	01/05/2020	31/08/2021	NCT04400838	Not yet recruiting
I/II	Sinovac vaccine	Inactivated	422	≥60	Yes	Double-blind, dose-finding	China	20/05/2020	20/07/2020	NCT04383574	Not yet recruiting
I	Novavax SARS-CoV-2 rS	Protein subunit	131	18-59	Yes	Observer-blind, dose-finding	Australia	25/05/2020	31/12/2020	NCT04368988	Recruiting
II	Moderna mRNA-1273	RNA	600	≥18	Yes	Observer-blind, dose-finding	USA	25/05/2020	31/03/2021	NCT04405076	Recruiting
I	Clover SCB-2019	Protein subunit	150	≥18	Yes	Double-blind, dose-finding	Australia	20/06/2020	20/10/2020	NCT04405908	Not yet recruiting
I/II	Chinese Academy of Medical Science vaccine	Inactivated	942	18-59	Yes	Double-blind, dose-finding	China	15/05/2020	30/09/2020	NCT04412538	Recruiting

**Live-attenuated vaccine:** live-attenuated vaccines use an altered version of SARS-CoV-2 so that it is less virulent (Table 2). These vaccines are very effective, and a single dose is often enough to induce long-lasting immunity. Serum Institute of India has partnered with US-based clinical-stage biotechnology company Codagenix to co-develop a live-attenuated vaccine against the coronavirus. Viruses will then be grown and tested in vivo by contracted laboratories suitable for containment, prior to testing in clinical trials [12]. Griffith University is working with Indian Immunologicals Limited to develop a live attenuated vaccine using a codon de-optimization technology to change the virus´s genome and decrease the replication efficiency in human cells [13]. The German Center for Infection Research is working on an attenuated virus (MVA: modified vaccinia virus Ankara), which had previously been used in a smallpox eradication vaccination campaign [14].

**Table 2:** live attenuated and inactivated COVID-19 vaccine candidates, WHO landscape (as of 09 June 2020)

Live attenuated COVID-19 vaccine candidates		
Vaccine type	Developer	Development Stage
Deoptimized live-attenuated	Serum Institute of India; Codagenix	Pre-clinical
Deoptimized live-attenuated	Indian Immunologicals Ltd; Griffith University	Pre-clinical
Live-attenuated measles virus	DZIF – German Center for Infection Research	Pre-clinical
**Inactivated COVID-19 vaccine candidates**		
Inactivated + alum	Sinovac/Dynavax	Phase 1 / 2 NCT04383574; NCT04352608
Inactivated	Wuhan Institute of Biological Products; Sinopharm	Phase 1 / 2 ChiCTR2000031809
Inactivated	Beijing Institute of Biological Products; Sinopharm	Phase 1 / 2 ChiCTR2000032459
Inactivated	Institute of Medical Biology, Chinese Academy of Medical Sciences (CAMS)	Phase 1 NCT04412538
Inactivated	Beijing Minhai Biotechnology Co., Ltd	Pre-clinical
Inactivated	Osaka University/ BIKEN/ NIBIOHN	Pre-clinical
Inactivated+CpG 1018	Sinovac/Dynavax	Pre-clinical
Inactivated+CpG 1018	Valneva/Dynavax	Pre-clinical
Inactivated	Research Institute for Biological Safety Problems, Kazakhstan	Pre-clinical

**Inactivated vaccine:** inactivated (or killed) vaccines consist of pathogens inactivated through physical, chemical, or biological means (Table 2). Beijing-based vaccine manufacturer, Sinovac´s candidate vaccine-called CoronaVac-was tested in 743 healthy volunteers between 18 and 59 years old, including 143 participants in Phase 1 and 600 in Phase 2. The vaccine induced neutralizing antibodies in over 90% of volunteers after receiving two doses, two weeks apart. Phase 3 clinical trials are expected to be conducted both within China and in countries outside China [15]. Sinopharm´s vaccine candidate, called BBIBP-CorV, induced neutralizing antibodies against SARS-CoV-2 in rodents, rabbits, and monkeys [16]. China´s Institute of Medical Biology candidate is in Phase 1 while the rest are in pre-clinical [3].

**Viral vector vaccine:** a vector is another virus that is not harmful and acts as the delivery system to carry antigens to the immune system. Scientists design a vector to carry only a small part of the SARS-CoV-2 genetic material so that it cannot cause infection. Once inside the body, the genetic material is converted to protein (Table 3). The advantages of viral vectors are: 1) high efficiency gene transduction; 2) highly specific delivery of genes to target cells; 3) induction of robust immune responses and increased cellular immunity [17]. This technology uses either live (replicating but attenuated) or non-replicating vectors. A growing number of viruses have been used as platforms to make experimental vaccines and for SARS-CoV-2, replicating viral vectors used include: yellow fever, measles, horsepox, influenza, Vesicular Stomatitis Virus, and Newcastle Disease Virus. Non-replicating viral vectors include: adenovirus, Modified Vaccinia Ankara (MVA), influenza, parainfluenza, and rabies [3].

**Table 3:** viral vector COVID-19 vaccine candidates, WHO landscape (as of 09 June 2020)

Vaccine candidate	Developer	Development Stage
**Replicating viral vector COVID-19 vaccine candidates**		
Replicating horsepox vector	Tonix Pharma/Southern Research	Phase 1 NCT04412538
Replicating YF17D vector	KU Leuven; UZ Leuven	Pre-clinical
Replicating measles vector	Zydus Cadila	Pre-clinical
Replicating measles vector	Institut Pasteur / Themis / Pittsburg Center for Vaccine Research / Merck	Pre-clinical
Replicating measles vector	FBRI SRC VB VECTOR, Rospotrebnadzor, Koltsovo	Pre-clinical
Attenuated influenza virus backbone (intranasal)	BiOCAD and IEM	Pre-clinical
Recombinant vaccine based on Influenza A virus	FBRI SRC VB VECTOR, Rospotrebnadzor, Koltsovo	Pre-clinical
Influenza expressing an antigenic portion of S protein	Fundação Oswaldo Cruz and Instituto Buntantan	Pre-clinical
M2-deficient single replication (M2SR) influenza vector	UW–Madison / FluGen / Bharat Biotech	Pre-clinical
Influenza vector expressing RBD	University of Hong Kong	Pre-clinical
Replication-competent VSV chimeric virus technology (VSVΔG)	IAVI / Merck	Pre-clinical
Vesicular Stomatitis Virus	University of Western Ontario	Pre-clinical
Vesicular Stomatitis Virus	FBRI SRC VB VECTOR, Rospotrebnadzor, Koltsovo	Pre-clinical
Newcastle disease virus vector	Intravacc / Wageningen Bioveterinary Research/Utrecht Univ	Pre-clinical
**Non-replicating viral vector COVID-19 vaccine candidates**		
ChAdOx1-s	University of Oxford / Astra Zeneca	Phase 1 / 2 2020-001072-15 Phase 2b / 3 2020-011228-32
Adenovirus Type 5 Vector	CanSino Biological Inc. / Beijing Institute of Biotechnology	Phase 1 ChiCTR2000030906 Phase 2 ChiCTR2000031781
Adeno-associated virus vector (AAVCOVID)	Massachusetts Eye and Ear / Massachusetts Gen Hospital / AveXis	Pre-clinical
MVA encoded VLP	GeoVax/ BravoVax	Pre-clinical
AD26 (alone or with MVA boost)	Janssen Pharmaceutical Companies	Pre-clinical
Replication defective Simian Adenovirus (GRAd)	ReiThera / LEUKOCARE / Univercells	Pre-clinical
MVA-S encoded	DZIF – German Center for Infection Research	Pre-clinical
MVA-S encoded	IDIBAPS-Hospital Clinic, Spain	Pre-clinical
Adenovirus-based NasoVAX expressing S-protein	AltImmune	Pre-clinical
[E1-, E2-, E3-] hAd5-COVID19-Spike/Nucleocapsid	ImmunityBio, Inc.; NantKwest, Inc.	Pre-clinical
Ad 5 (GREVAX ™) platform	Greffex	Pre-clinical
Oral Ad 5 S	Stabilitech Biopharma Ltd	Pre-clinical
Adenovirus-based + HLA-matched peptides	Valo Therapeutics Ltd	Pre-clinical
Oral vaccine platform	Vaxart	Pre-clinical
MVA-S encoded	Centro Nacional Biotechnologia (CNB-CSIS), Spain	Pre-clinical
Dendritic cell based vaccine	University of Manitoba	Pre-clinical
Parainfluenza virus 5 (PIV5)-based vaccine expressing the S protein	University of Georgia; University of Iowa	Pre-clinical
Recombinant deactivated rabies virus containing S1	Bharat Biotech; Thomas Jefferson University	Pre-clinical
Inactivated flu-based vaccine + adjuvant	National Center for Genetic Engineering & Biotechnology (BIOTEC) / GPO, Thailand	Pre-clinical

China´s CanSino Biologics was the first company in the world to begin a clinical study of a SARS-CoV-2 vaccine. Less than 10 weeks later, the company published the Phase 1 trial data. The vaccine candidate, using a genetically engineered adenovirus vector to deliver the gene that encodes the SARS-CoV-2 spike protein into human cells. CanSino measured neutralizing antibodies concentrations in subjects and found that 75% of people who received the high dose and 50% of those who received a medium or low dose developed levels of neutralizing antibodies considered high by the researchers [18]. AZD1222, developed by Oxford University´s Jenner Institute and the Oxford Vaccine Group, uses a replication-deficient chimpanzee viral vector based on an attenuated version of a common cold (adenovirus) virus that causes infections in chimpanzees and contains the genetic material of SARS-CoV-2 spike protein. The vaccine has gone through Phase 1 and is starting Phase 2/3 in England and Brazil [19].

**Protein subunit vaccine:** instead of the whole pathogen, subunit vaccines include only specific components or antigens that have been proven through pre-clinical studies to stimulate the immune system (Table 4) [20]. Including only certain antigens in the vaccine can minimize side effects but it usually requires the addition of adjuvants to elicit a stronger immune response because antigens alone are not sufficient to elicit adequate long-term immunity [21]. There are several protein-based vaccine candidates (similar to 50) [3]. The candidates furthest along in clinical trials are the one made by Shenzhen Geno-Immune Medical Institute (COVID-19 aAPC) and Novavax´s protein subunit vaccine (NVX CoV2373). COVID-19 aAPC vaccine uses a lentivirus to construct artificial antigen-presenting cells (APCs) to present structural and nonstructural SARS-CoV-2 antigens and is administered in three doses [22]. The Phase 1/2 clinical trial of the Novavax, supported by the Coalition for Epidemic Preparedness Innovations (CEPI), is being conducted in two parts. Phase 1, conducted in Australia, is a randomized, observer-blinded, placebo-controlled trial designed to evaluate the immunogenicity and safety of both adjuvanted with Matrix M and unadjuvanted. The protocol´s two-dose trial regimen assesses two dose sizes (5 and 25 micrograms) with Matrix M and without. Phase 2, to be conducted in multiple countries, including the United States, will assess immunity, safety and COVID-19 disease reduction in a broader age range [23]. Most protein-based vaccine candidates are targeting the Spike (S) protein, while others are targeting the receptor binding domain (RBD). The candidate from the University of Queensland uses a peptide frozen into prefusion conformation via a molecular clamp. This potentially promotes a strong neutralizing antibody response, but earlier study on Respiratory syncytial virus (RSV) showed the technology induced an antibody response that was robust but not neutralizing [24].

**Table 4:** protein subunit COVID-19 vaccine candidates, WHO landscape (as of 09 June 2020)

Vaccine candidate	Developer	Development Stage
COVID-19 artificial antigen-presenting cells (APCs)	Shenzhen Geno-Immune Medical Institute	Phase 1
Native like Trimeric subunit Spike Protein vaccine	Clover Biopharmaceuticals Inc. / GSK / Dynavax	Phase 1 NCT04405908
Full length recombinant SARS-CoV-2 glycoprotein nanoparticle + Matrix M adjuvant	Novavax	Phase 1 / 2 NCT04368988
Adjuvanted microsphere peptide	VIDO-InterVac, University of Saskatchewan	Pre-clinical
Adjuvanted recombinant protein (RBD-Dimer)	Anhui Zhifei Longcom Biopharmaceutical / Institute of Microbiology, Chinese Academy of Sciences	Pre-clinical
Adjuvanted protein subunit (RBD)	Biological E Ltd	Pre-clinical
Capsid-like protein	AdaptVac (PREVENT-nCoV consortium)	Pre-clinical
COVID-19 XWG-03 truncated S (spike) proteins	Innovax / Xiamen University / GSK	Pre-clinical
Drosophila S2 insect cell expression system	ExpreS2ion	Pre-clinical
gp-96 backbone	Heat Biologics / University of Miami	Pre-clinical
Ii-Key peptide	Generex / EpiVax	Pre-clinical
Microneedle arrays S1 subunit	University of Pittsburgh	Pre-clinical
Molecular clamp stabilized Spike protein	University of Queensland / GSK / Dynavax	Pre-clinical
Nanoparticle vaccine	LakePharma, Inc.	Pre-clinical
OMV-based vaccine	Quadram Institute Biosciences; BiOMViS Srl / University of Trento	Pre-clinical
OMV-based subunit	Intravacc / Epivax	Pre-clinical
OMV-based peptide	Intravacc / Epivax	Pre-clinical
Oral E. coli-based protein expression system of S and N proteins	MIGAL Galilee Research Institute	Pre-clinical
Orally delivered, heat stable subunit	Applied Biotechnology Institute, Inc.	Pre-clinical
Peptide	Vaxil Bio; Flow Pharma Inc; FBRI SRC VB VECTOR, Rospotrebnadzor, Koltsovo	Pre-clinical
Peptide antigens formulated in LNP	ImmunoVaccine Inc.	Pre-clinical
Peptides derived from Spike protein	Axon Neuroscience SE	Pre-clinical
Protein subunit	University of San Martin and CONICET, Argentina ; MOGAM Institute for Biomedical Research, GC Pharma	Pre-clinical
Protein Subunit EPV-CoV-19	EpiVax	Pre-clinical
RBD-based	Neovii / Tel Aviv University; Kentucky Bioprocessing, Inc.; Baylor College of Medicine	Pre-clinical
Recombinant protein	Yisheng Biopharma	Pre-clinical
Recombinant S protein in IC-BEVS	Vabiotech	Pre-clinical
Recombinant protein, nanoparticles (based on S-protein and other epitopes)	St. Petersburg Research Institute of Vaccines & Serums	Pre-clinical
Recombinant spike protein with Advax™ adjuvant	Vaxine Pty Ltd / Medytox	Pre-clinical
Recombinant S1-Fc fusion protein	AnyGo Technology	Pre-clinical
RBD protein fused with Fc of IgG + adjuvant	Chulalongkorn University/GPO, Thailand	Pre-clinical
S protein	WRAIR / USAMRIID; AJ Vaccines; Sanofi Pasteur / GSK	Pre-clinical
S protein + adjuvant	National Institute of Infectious Disease, Japan	Pre-clinical
S peptide	EpiVax / University of Georgia	Pre-clinical
Subunit vaccine	FBRI SRC VB VECTOR, Rospotrebnadzor, Koltsovo	Pre-clinical
Subunit protein, plant produced	iBio/ CC-Pharming	Pre-clinical
Synthetic Long Peptide Vaccine candidate for S and M proteins	OncoGen	Pre-clinical
Structurally modified spherical particles of the tobacco mosaic virus (TMV)	Lomonosov Moscow State University	Pre-clinical
Spike-based	University of Alberta	Pre-clinical
Spike-based (epitope screening)	ImmunoPrecise	Pre-clinical
S-2P protein + CpG 1018	Medigen Vaccine Biologics Corp / NIAID / Dynavax	Pre-clinical
VLP-recombinant protein + adjuvant	Osaka University / BIKEN / National Institutes of Biomedical Innovation, Japan	Pre-clinical

**Vaccines based on virus-like particles (VLPs):** Virus-like particles (VLPs) are structures resulting from self-assembly of virus proteins without a nucleic acid genome or a lipid envelope (Table 5). VLPs have structural and antigenic similarity with the parental virus and some have proven to be successful as vaccines against virus infection [25]. The human immune system recognizes and interacts with VLPs on the basis of two major characteristics: size and surface geometry [26].

**Table 5:** VLP-based vaccine candidates, WHO landscape (as of 09 June 2020)

Vaccine type	Developer	Development Stage
VLP + Adjuvant	Mahidol University/ The Government Pharmaceutical Organization (GPO)	Pre-clinical
VLP, lentivirus and baculovirus vehicles	Navarrabiomed, OncoImmunology group	Phase 1 NCT04412538
Cucumber Mosaic Virus VLP	Saiba AG; AGC Biologics	Pre-clinical
Plant-derived VLP	Medicago Inc.	Pre-clinical
ADDomer™ multiepitope display	Imophoron Ltd; Bristol University's Max Planck Centre	Pre-clinical
VLP	Doherty Institute	Pre-clinical
VLP	OSIVAX	Pre-clinical
envelope virus like particles (eVLP)	ARTES Biotechnology	Pre-clinical
VLPs peptides / whole virus	University of Sao Paulo	Pre-clinical
Spike-based (epitope screening)	ImmunoPrecise	Pre-clinical

**DNA vaccines:** DNA vaccination involves the direct introduction into appropriate tissues of a plasmid containing the DNA sequence encoding the antigen or antigens for which an immune response is desired (Table 6) [27]. The DNA encoding the target molecule is introduced via a plasmid or viral vector or cell line, in which DNA is expressed and translated into protein. The injected DNA is a plasmid plus a promoter that provides immunogenic protein synthesis [28]. DNA vaccines can stimulate both humoral and cellular immunity and do not require maintenance under the usual conditions for traditional vaccine (+2°C to +8°C). In addition, unlike live attenuated vaccines, the risks arising from a potential inadequate attenuation are non-existent for DNA vaccines [29].

**Table 6:** DNA and RNA vaccine candidates, WHO landscape (as of 09 June 2020)

Vaccine type	Developer	Development Stage
**DNA vaccine candidates**		
DNA plasmid vaccine with electroporation	Inovio Pharmaceuticals	Phase 1 NCT04336410
bacTRL-Spike	Symvivo	Phase 1 NCT04334980
DNA vaccine (GX-19)	Genexine Consortium	Pre-clinical
DNA plasmid vaccine with electroporation	Karolinska Institute / Cobra Biologics (OPENCORONA Project)	Pre-clinical
DNA plasmid vaccine	Osaka University / AnGes / Takara Bio	Pre-clinical
DNA vaccine	Takis / Applied DNA Science / Evvivax	Pre-clinical
DNA plasmid, needle-free delivery	Immunomic Therapeutics, Inc. / EpiVax, Inc. / PharmaJet	Pre-clinical
DNA plasmid vaccine	Zydus Cadila	Pre-clinical
DNA vaccine	BioNet Asia	Pre-clinical
DNA vaccine	Entos Pharmaceuticals	Pre-clinical
**RNA vaccine candidates**		
LNP-encapsulated mRNA	Moderna / National Institute of Allergy and Infectious Diseases	Phase 1 NCT04283461; Phase 2 NCT04405076
3 LNP-encapsulated mRNAs	BioNTech / Fosun Pharma / Pfizer	Phase 1 / 2 2020-001038-36; NCT04368728
LNP-mRNA	Translate Bio/Sanofi Pasteur CanSino Biologics / Precision NanoSystems	Pre-clinical
LNP-encapsulated mRNA cocktail encoding VLP	Fudan University / Shanghai Jiao Tong University / RNACure Biopharma	Pre-clinical
LNP-encapsulated mRNA encoding RBD	Fudan University / Shanghai Jia Tong University / RNACure Biopharma	Pre-clinical
Replicating defective SARS-CoV-2 derived RNA	Centro Nacional Biotecnologia (CNB-CSIC), Spain	Pre-clinical
LNP-encapsulated mRNA	University of Tokyo / Daiichi-Sankyo	Pre-clinical
Liposome-encapsulated mRNA	BIOCAD	Pre-clinical
Several mRNA candidates	RNAimmune, Inc.	Pre-clinical
mRNA	FBRI SRC VB VECTOR, Rospotrebnadzor, Koltsovo	Pre-clinical
mRNA	China CDC / Tongji University / Stermina	Pre-clinical
mRNA	Arcturus / Duke-NUS Singapore	Pre-clinical
saRNA	Imperial College London	Pre-clinical
mRNA	CureVac	Pre-clinical
mRNA in an intranasal delivery system	eTheRNA	Pre-clinical
mRNA	Greenlight Biosciences	Pre-clinical
mRNA	Institut d'Investigacions Biomèdiques August Pi i Sunyer IDIBAPS-Hospital Clinic, Spain	Pre-clinical

INO-4800 is being developed by Inovio Pharmaceuticals and its partner Beijing Advaccine Biotechnology, with the support of a Coalition for Epidemic Preparedness Innovations (CEPI) grant. INOVIO has extensive experience working with coronaviruses and has a Phase 2a vaccine for a related coronavirus that causes Middle East Respiratory Syndrome (MERS). INO-4800 is using CELLECTRA 3PSP, a portable, hand-held delivery device that delivers a short electrical pulse to open small pores int the cell, enabling the plasmid to enter. Once inside, the cell uses the plasmid to produced coded antigens, which trigger an immune response. INO-4800 entered Phase 1 in April 2020. Participants will receive two doses of INO-4800 every four weeks and initial safety and immune response data from the study are expected by 3rd quarter of 2020. Inovio has partnered with Advaccine and the International Vaccine Institute to advance Phase 2/3 clinical trials in China and South Korea, respectively [30].

**RNA vaccines:** there are over a dozen mRNA COVID-19 vaccine candidates and 2 are in clinical phase. mRNA-1273, from the Vaccine Research Center at the National Institute of Allergy and Infectious Diseases NIAID and the biotech Moderna, is a novel lipid nanoparticle (LNP)-encapsulated mRNA vaccine against the COVID-19 encoding for a prefusion stabilized form of the Spike (S) protein (Table 6). Like the DNA vaccine, the mRNA technology injects snippets of genetic code into a person´s muscle so that the muscle cells, in theory, start producing the viral protein themselves. The Phase 1 open-label, dose-ranging trial study (NCT04283461)) evaluated the safety and immunogenicity of three dose levels of mRNA-1273 (25, 100, 250 μg) administered on a two-dose vaccination schedule, given 28 days apart. An analysis of the response in eight individuals showed that those who received a 100 microgram dose and people who received a 25 microgram dose had levels of protective antibodies to fend of the virus that exceeded those found in the blood of people who recovered from COVID-19, the illness caused by the coronavirus [31]. mRNA-127 is currently in a Phase II clinical trial, which will enroll 600 healthy participants aged 18 and above. Phase 3 trials will begin in July and will primarily study the efficacy of the vaccine in preventing symptomatic COVID-19 disease and secondarily, the prevention of severe cases of COVID-19 which require hospitalization [32]. Pfizer and BioNTech´s COVID-19 mRNA vaccine program, BNT162, started Phase 1 clinical trials in May. The Phase 1/2 study is designed to determine the safety, immunogenicity, and optimal dose level of four mRNA vaccine candidates evaluated in a single, continuous study. The dose level escalation portion (Stage 1) of the Phase 1/2 trial in the U.S. will enroll up to 360 healthy subjects into two age cohorts (18-55 and 65-85 years of age [33].

**Existing live attenuated vaccines for other diseases:** an increasing body of evidence suggests that live vaccines can induce broader protection beyond the specific protection against the targeted pathogen. These non-specific effects (also called “heterologous effects” or “off-target effects”) likely occur by inducing interferon and other innate immunity. Non-specific effects have been discussed in the past. In 2013, a working group organized by the WHO systematically evaluated the evidence for non-specific effects of Bacillus Calmette-Guérin (BCG), measles and DTP (diphtheria, pertussis, tetanus) vaccines. The following year, the WHO reviewed the evidence and concluded that the findings merit further research [34]. The stimulation of innate immunity by BCG or oral polio vaccine (OPV) could provide temporary protection against COVID-19. BCG is already being studied by several groups in different countries. For all live vaccines (BCG, oral polio vaccine, measles), the theory is that they induce protection against several infections (apart from the ones they are supposed to work against) by long-term boosting of innate immune responses (called “trained immunity”). When the immune systems of people who had the BCG vaccine were compared to those who have not, it´s been shown that the immune cells that first respond to disease in BCG vaccinated people are more alert and ready to act on a potential threat [35]. Researchers in The Netherlands and Greece [36] have started a clinical trial using BCG. Other live attenuated TB vaccines candidates in clinical trials [37] are VPM1002, [38] derived from BCG, or MTBVAC derived from *M. tuberculosis*. Like BCG, these could show non-specific effects and could be candidates to be studied for their protection against COVID-19. OPV has been shown to reduce infection-related hospitalization in developed countries by providing protection against unrelated pathogens. With a proven safety profile, there is enough scientific justification to evaluate OPV for anti-viral protection against SARS-CoV-2 [39]. An analysis of the effect of annual and biannual national OPV immunization campaigns showed that they reduced all-cause mortality by 19%, with each subsequent campaign adding a further 13% reduction [40] suggesting that repeated immunization could have additive protective effects.

**Expert commentary:** vaccines are preventive or therapeutic interventions that dramatically reduce morbidity and mortality caused by infectious diseases. They are clinically simple but immunologically complex. The pressure to develop a COVID-19 vaccine is huge. But its development without fully understanding the kinetics of immune responses involved in the disease and the safety risks of the vaccine could bring unwarranted setbacks-now and in the future. In addition, SARS-CoV-2 might mutate in ways that would make previously effective vaccines useless. A great many steps have to be taken in the development of any vaccine. With COVID-19, there are added complexities given that its severity appears be different across gender and age. There´s also evidence that it might be mutable and that it has different strains. Then there is the fact that it is very new, which means there´s still limited knowledge about immune responses to SARS-CoV-2. In addition, a multiplicity of disciplines must be involved. A safe and effective vaccine will not be developed without detailed understanding of host-pathogen interaction. This is happening in the trials that are being currently run. What this adds up to is that a safe and efficient COVID-19 vaccine might not be realized soon. Most experts think a vaccine is likely to become widely available by mid-2021, about 12-18 months after the new virus, known officially as SARS-CoV-2, first emerged. That would be a huge scientific feat and there are no guarantees it will work. Four coronaviruses already circulate in human beings. They cause common cold symptoms and we do not have vaccines for any of them.

Coronaviruses display spicules (Spike protein or S protein) which they use to attach to receptors in human cells. Many of the vaccine candidates are targeting the S proteins as these are well recognized by the human immune system. This is true for all strains of coronavirus, including SARS-CoV-1, MERS-CoV, and SARS-CoV-2 responsible for COVID-19 [41]. The scientific community has learned a lot about COVID-19 considering that the virus and the disease only emerged in early 2020 but the immune mechanism is still not well understood particularly on how the immune system reacts to the virus although severity stems from inappropriate, excessive and/or inadequate immune responses. A major challenge of these vaccine candidates will be immune enhancement - discovered in the 1960s when a vaccine candidate for respiratory syncytial virus (RSV) was tested which showed that the disease worsened after vaccinated children were exposed to the virus, with 2 mortalities. Decades ago, animal vaccines developed against another coronavirus, feline infectious peritonitis virus, increased cats´ risk of developing the disease caused by the virus [42]. Similar phenomena have been seen in animal studies for other viruses, including the coronavirus that causes severe acute respiratory syndrome (SARS) [43]. The mechanism that causes this is not fully understood and is one of the difficulties of successful development of a coronavirus vaccine.

Scientific research landscape has a pattern where emergence of novel pathogens causing an outbreak leads to an increase in research investment but when the outbreak dies down, priorities change and interest in research stops. Funding for this kind of research should rest with governments and non-profits because for-profit pharmaceutical companies do not have interest to fund projects that will not have commercial potential. Progress was made in the West Africa Ebola outbreak that ended in 2016. It spurred the creation of the Coalition for Epidemic Preparedness Innovations (CEPI) [44], a private-public partnership based in Norway and funded in part by the Bill and Melinda Gates Foundation. Funding is one of the major factors for the unprecedented speed in the development of vaccines for COVID-19. The often mentioned “12-18 months” (i.e. in 2021) is the bare minimum amount of time needed to develop a vaccine-this is possible only if all the phases in the clinical trials are successful. The inactivated mumps vaccine, considered the fastest ever approved, took three years to develop from identification of the pathogen and collecting viral samples to licensing. Vaccine clinical trials involve testing healthy individuals and following up after a specific amount of time to check for safety and efficacy. Phase 1 for safety lasts between 1-2 years; Phase 2 to further demonstrate safety and some efficacy lasts between 2-3 years and Phase 3 for safety and efficacy in natural disease conditions lasts between 5-10 years. Regulators must continue to require vaccine developers to check for potentially harmful responses in animal studies. They must also carefully assess the volunteers for the presence of antibodies against any coronaviruses before enrolling them in safety trials.

Given the uncertainty in defining a correlate of protection, a vaccine candidate that generates both humoral and cellular immune responses is desirable, and this ideally should be shown by the vaccine candidates. It is also necessary to be clear on the objective of the vaccine. A vaccine capable of protecting against the complications of COVID-19 is already a good vaccine. Induction of total immunity (called “sterilizing immunity”) is a high bar for a vaccine. Inducing protective immune response in healthy volunteers is already a challenge but it is expected to be even more challenging in people with weakened immune system by old age, obesity, illness or medical treatments that slow down immune defences. Vaccines with effective adjuvants are often needed to protect these vulnerable populations. The U.S. Food and Drug Administration (FDA) has signalled that when responding to an urgent public health situation such as novel coronavirus, regulatory flexibility and accelerated testing schedules should be considered. One option to accelerate timeline for vaccine development is approval under the FDA´s Animal Rule [45] established to facilitate approval of new products for life-threatening conditions when traditional trials in humans are unethical or impractical. Vaccine developers are still required to conduct routine animal testing to make sure the vaccine itself is not toxic and induces protection from the virus. With anti-government sentiments and the anti-vaccine movement, the urgency of vaccines should be weighed carefully with safety risks. Rushing vaccines without fully understanding certain phenomena, such as immune enhancement, could result to unwarranted setbacks and further aggravate anti-science.

## Conclusion

The unprecedented morbidity and mortality from the current COVID-19 pandemic has challenged every aspect of our global ability to effectively detect, respond to, and control such a rapidly emerging infectious disease. In response to this urgent global health crisis, a massive effort is under way to develop vaccines for coronavirus within months and make it available to save lives. Several candidate vaccines strategies are being investigated in laboratories of universities and companies in many parts of the world. Of the over 130 vaccine candidates, 17 are already in clinical trials, while the others are in various stages of preclinical development. Each of the different vaccine platforms available, traditional or novel, is currently being explored. Some platforms, such as DNA and RNA vaccines, have not produced licensed vaccines but may prove to be the first one to reach the finish line. Three vaccine candidates, one each from the US, UK, and China, have completed Phase I. While vaccine efficacy of the candidates is still under evaluation, there have been few or no adverse reactions in humans. Not a single vaccine has been approved for any other coronavirus so far, and there is no guarantee that a successful SARS-CoV-2 vaccine will be available soon. Robust and well-designed trials in populations with ongoing outbreaks in multiple locations and international collaborations are necessary to develop safe and effective COVID-19 vaccines.
